# Study on Extraction Separation of Thioarsenite Acid in Alkaline Solution by ${\text{CO}}_{3}^{2-}$-Type Tri-n-Octylmethyl-Ammonium Chloride

**DOI:** 10.3389/fchem.2020.592837

**Published:** 2021-01-20

**Authors:** Kang Yan, Liping Liu, Hongxing Zhao, Lei Tian, Zhifeng Xu, Ruixiang Wang

**Affiliations:** ^1^School of Metallurgical Engineering, Jiangxi University of Science and Technology, Ganzhou, China; ^2^Henan Yuguang Gold and Lead Group Co., Ltd., Jiyuan, China; ^3^President Office, Jiangxi College of Applied Technology, Ganzhou, China

**Keywords:** extraction separation, thioarsenite acid, alkaline solution, CO32--type, tri-n-octylmethyl-ammonium chloride

## Abstract

To overcome the problem of arsenic separation and enrichment from an alkaline leaching solution in arsenic-containing dust, a ${\text{CO}}_{3}^{2-}$-type tri-n-octylmethyl-ammonium chloride (TOMAC) method for extracting thioarsenite is proposed in this paper. Considering an alkaline leaching solution as the research object, after vulcanization pretreatment, TOMAC transformation and organic phase saturated extraction capacity were measured, and the extraction mechanism was preliminarily studied. First, Cl^−^-type quaternary ammonium salt was effectively transformed to ${\text{HCO}}_{3}^{-}$-type by treating organic phase with saturated NaHCO_3five_ times. TOMAC was effectively transformed from ${\text{HCO}}_{3}^{-}$ to ${\text{CO}}_{3}^{2-}$ type by alkaline washing with 1.0 mol/l NaOH solution; this washing was repeated thrice. Thereafter, the effects of organic phase composition, phase ratio, extraction time, and temperature on the extraction and separation of arsenic were investigated. The results show that under the conditions of 30% ${\text{CO}}_{3}^{2-}$-type TOMAC + 15% sec-octanol + 55% sulfonated kerosene, V_O_/V_A_ = 1/1, and 5 min extraction at room temperature, the single-stage extraction rate of As^III^ is 85.2%. The As^III^ concentration in raffinate can be reduced to less than 1.33 × 10^−3^ mol/l by four-stage countercurrent extraction, and the extraction rate of As^III^ can exceed 98.4%.

## Introduction

Arsenic and its compounds are volatile. Arsenic is mainly concentrated in smelting dust, owing to the high-temperature volatilization, airflow movement, and mechanical inclusion during the smelting process of heavy non-ferrous metals, such as copper, lead, and zinc (Christof Lanzerstorfer, 2016). Arsenite or subarsenite is formed by the collision and adsorption of arsenic with lead, antimony, zinc, and other elements in high-temperature gas. The content of arsenic has a wide range from 10 to 40% (Jarošíková et al., 2018). Besides arsenic, high arsenic dust also contains a large amount of valuable metals, such as copper, lead, zinc, tin, and indium, which have high economic value (Asanov et al., 2016). High arsenic dust has the environmental characteristics of large production and high toxicity.

The arsenic content in smelting dust is volatile, which has the characteristics of large production and high toxicity. Therefore, it is necessary to efficiently separate arsenic from smelting dust for further recycling (Ermolin et al., 2019). There have been many studies on arsenic removal from smelting dust, including roasting, leaching, and combined pyro-hydrometallurgical processes. Arsenic removal by calcination serves mainly to volatilize arsenic in the form of arsenic trioxide in materials containing arsenic at high temperature, separate it from other valuable metals, and then obtain crude arsenic trioxide products through condensation and dust collection (Montenegro et al., 2013).

Arsenic is extracted from copper dust by leaching process; leaching can be classified as hot water leaching, acid leaching, and alkaline leaching according to the properties of leaching solution (Guo et al., 2016). The following methods for separating and enriching arsenic from leaching liquid include evaporation concentration crystallization, lime precipitation, ferric salt precipitation, sodium sulfide precipitation, adsorption, and solvent extraction (Hoffmann, 1993; Sanchez de la Campa et al., 2008; Morales et al., 2010). Sahu et al. investigated the acid leaching of copper from the soot of electrostatic precipitator (ESP) liner used in a copper smelter plant. Results showed that the acid concentration of 1.5 M and pulp density of 20% was found to be optimum, and the leaching efficiency of copper was 97% at 97°C (Sahu et al., 2012).

To achieve the resource utilization of flue dust, most copper smelters send flue dust and copper concentrate directly back to the smelting system, which greatly increases the content of impurities (especially arsenic) in the flash-smelting furnace. The Kosaka smelter in Japan has been operated with the open-process hydrometallurgical treatment for flue dust since 1975. This process involves recovering copper and zinc from the flue dust leaching solution and lead from the leaching slag. Similarly, most studies in this area have adopted the combined process of “hydro and pyro metallurgy” to treat the flue dust, i.e., to leach copper and zinc using water or dilute sulfuric acid and recover lead from the leaching slag through reduction smelting.

Karimov et al. studied the sulfuric acid leaching of dust left over from the reduction smelting at the Middle Ural Copper Smelter. The results showed that the optimum parameters for leaching dust were a temperature of 60°C and an initial acid concentration of 25 g/dm^3^. Performing the leaching operation with these parameters maximizes the yields of arsenic, copper, and zinc (98% As, 39% Cu, and 82% Zn) (Karimov and Naboichenko, 2016). Yang et al. reported the recovery of metals from copper smelting dust via H_2_SO_4_ and H_2_O_2_ leaching. Under optimum conditions, the leaching efficiencies achieved for Cu, As, Fe, Cd, and Zn were 93.4, 94.2, 39.7, 98.1, and 90.7%, respectively (Yang et al., 2017). Liu et al. investigated the metal extraction from copper smelting dust using oxidation leaching and the control of potential technology. The results showed that under the conditions of an H_2_O_2_ dosage of 0.8 ml/g (redox potential is 429 mV), H_2_O_2_ feeding speed of 1.0 ml/min, initial H_2_SO_4_ concentration of 1.0 mol/l, initial HCl concentration of 1.0 mol/l, leaching temperature of 80°C, initial liquid-to-solid ratio of 5:1 ml/g, and leaching time of 1.5 h, copper and arsenic can be effectively leached from copper smelting dust, leaving residue as a suitable lead resource. The average leaching efficiencies of copper, arsenic, and iron were 95.27, 96.82, and 46.65%, respectively (Liu et al., 2018). Xu et al. studied pressure-leaching technology in the treatment of high-copper and high-arsenic dust. At a liquid-to-solid ratio (ml/g) of 5:1, a leaching temperature of 453 K, a retention time of 2 h, an initial sulfuric acid concentration of 0.74 mol/l, an oxygen partial pressure of 0.7 MPa, and an agitation speed of 500 r/min, 95% of copper, 99% of zinc, and only 6% of iron in the dust were leached, whereas ~20% of arsenic was also leached. The leaching technique was optimized further to restrain the leaching of arsenic through the addition of a small amount of ferrous iron into the leaching system [*c*(Fe(2+)) = 0.036 mol/l] (Xu et al., 2010).

While extracting arsenic from flue dust using dilute acid, significant dispersibility can be observed; ~50% of arsenic enters the solution, whereas the other half enters the slag. The chemical precipitation of arsenic in the leaching solution can result in a loss of valuable metals, whereas the landfilling and stockpiling of the arsenic slag can result in potential secondary pollution. Consequently, the separation and enrichment of arsenic using the traditional extraction process is low; therefore, it is not suitable for the resource utilization and harmless disposal of high arsenic flue dust.

In contrast, arsenic oxides and arsenates are easily soluble in alkaline medium, based on which some studies have proposed the use of alkaline leaching to achieve arsenic concentration in the water phase. Reynolds et al. carried out NaOH leaching on the pressure-leaching slag of copper smelting dust (arsenic and iron slag), reaching an arsenic leaching rate of 88.3% (Reynolds, 1981). Furthermore, the NaOH-Na_2_S alkaline leaching process for the oxidation leaching of high arsenic flue dust led to an arsenic leaching rate of greater than 90% (Liu et al., 2009). Rappas et al. adopted a two-stage alkaline leaching process to effectively separate arsenic from lead and bismuth (Rappas et al., 1990). In fact, for high arsenic materials (e.g., arsenic sulfide slag), alkaline leaching is equally effective in arsenic removal (Zheng et al., 2008).

**Figure 1:**
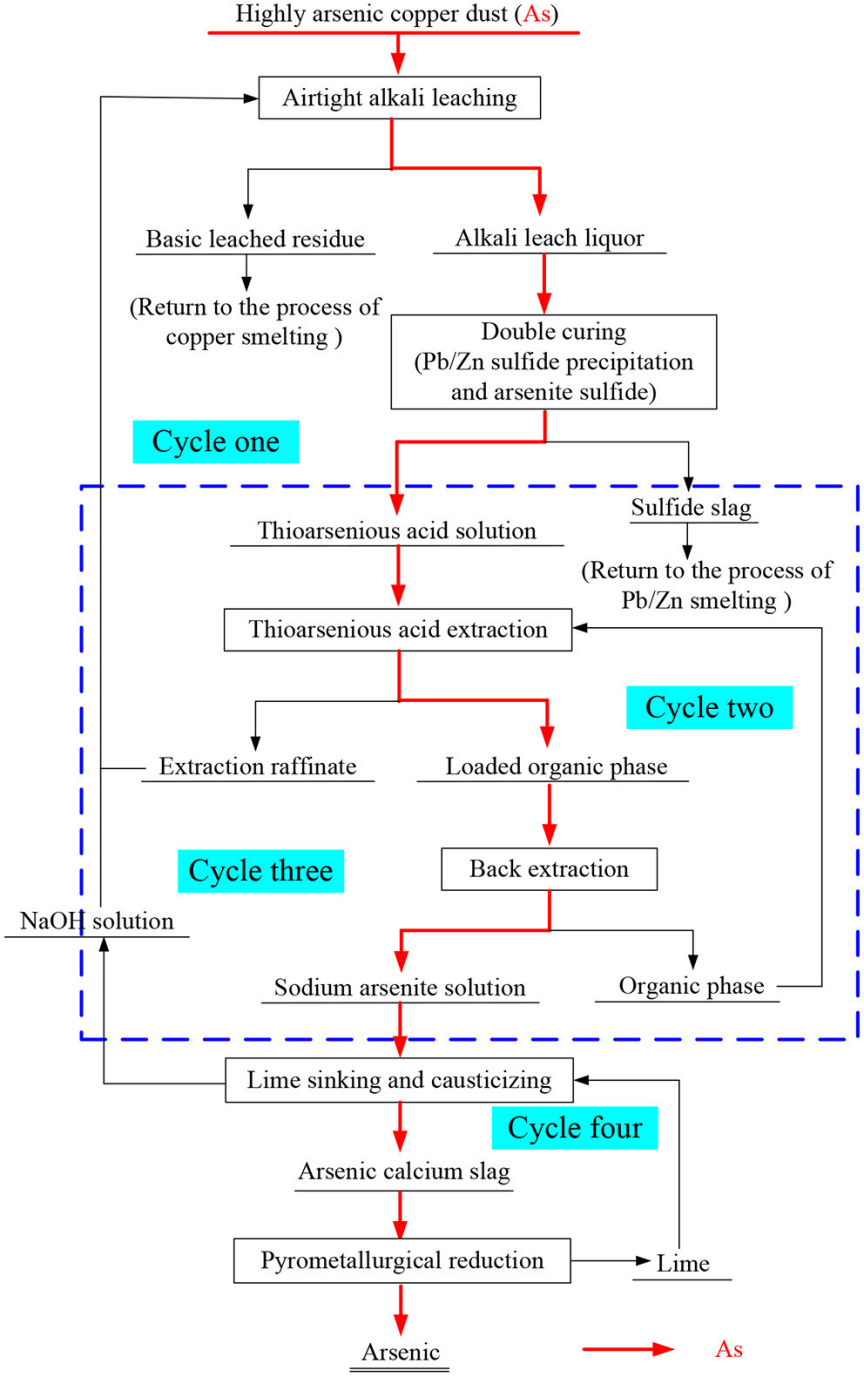
Process flow chart of “closed” alkali leaching double sulfurization coordination solvent extraction arsenic removal lime cascade arsenic deposition carbothermal reduction”.

Although the effective separation of arsenic and the treatment of valuable metals can be achieved by alkaline leaching, how to recycle the alkali liquor and enrich arsenic efficiently to facilitate a final harmless disposal are the key difficulties. Therefore, a new process has been proposed; this process involves closed alkaline leaching, double-sulfuration synergistic solvent extraction, de-arsenic–lime cascade precipitation, and arsenic–carbon thermal reduction for high arsenic copper smelting ash (as shown in Figure 1).

Based on previous experimental studies (Xu et al., 2016), the sulfurized products of arsenous acid under alkaline conditions are mainly HAsO_2_S^2−^ and ${\text{HAsOS}}_{2}^{2-}$; however, there are currently still some limitations on the extraction and stripping of thioarsenite in alkaline solutions. In alkaline media, arsenic exists in the form of thioarsenite anions, and quaternary ammonium salt is a strong base salt, which contains R_4_N^+^ groups to extract complex anion (Guan and Zhang, 2011). Therefore, herein, experiments on the extraction and stripping of thioarsenite in NaOH solutions were conducted using tri-n-octylmethyl-ammonium chloride (TOMAC) as the extractant and ${\text{CO}}_{3}^{2-}$ for anion conversion. This study provided solutions for the extraction and separation of arsenic in an alkaline medium and laid the theoretical foundation for the realization of a highly efficient process of arsenic separation, improvement in arsenic resource utilization, and establishment of a comprehensive recovery system of valuable metals.

## Experimental

### Materials

TOMAC was kindly supplied by Shanghai Titan Technology Co., Ltd. All the extractants were used without further purification and dissolved in sulfonated kerosene provided by Nanjing Runchuan Petrochemical Co. Ltd., China at the required concentrations. The thioarsenious acid solutions were prepared by dissolving NaAsO_2_ and Na_2_S·9H_2_O (Aladdin, Shanghai) in lye (NaOH) to the required concentration under different conditions of temperature and time. All the other reagents and chemicals used were of analytical reagent grade.

### Leaching Experiment

#### TOMAC Transformation From Cl^−^ to ${\text{CO}}_{3}^{\text{2}-}$-Type Experiment

**Figure 2:**
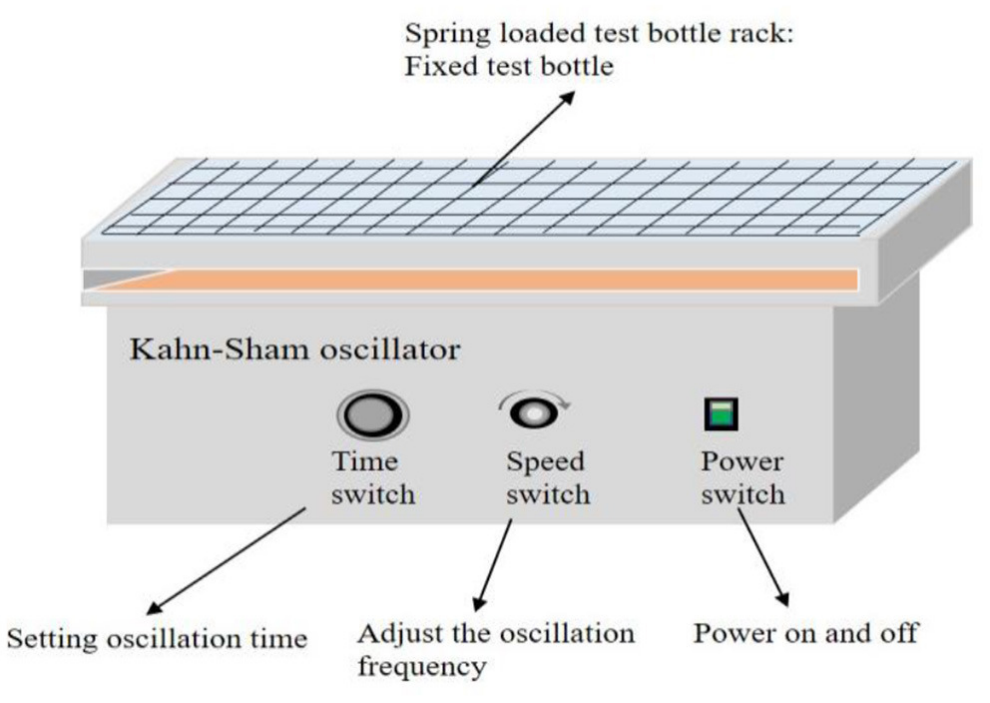
Construction of Kahn-Sham oscillator.

After the addition of a certain amount of TOMAC into a pear-shaped funnel, NaHCO_3_ saturated solution was added, V_O_/V_A_ = 1/1, and the conditions of water phase are as follows: the concentration of NaOH is 0.5 mol/l and the concentration of As^III^ is 9.69 × 10^−2^ mol/l. The first extractant transformation was completed after 10 min of mixing in a Kohn–Sham (KS) oscillator, and the construction is shown in Figure 2. Then, the organic phase and water phase were separated, and the concentration of Cl^−^ in the solution after transformation was analyzed to obtain the anion conversion rate of TOMAC from Cl^−^ to ${\text{HCO}}_{3}^{-}$ type. The second mixed organic phase transformation was conducted using NaOH solution at a certain concentration by the same experimental procedure as the first. After the second extractant transformation, the concentrations of ${\text{CO}}_{3}^{2-}$ and OH^−^ in the solution were analyzed again to obtain the anion conversion rate of TOMAC from ${\text{HCO}}_{3}^{-}$ to ${\text{CO}}_{3}^{2-}$ type.

#### Extraction of Thioarsenious Acid With ${\text{CO}}_{3}^{2-}$-Type TOMAC

${\text{CO}}_{3}^{2-}$-type TOMAC and thioarsenious acid solutions were mixed in a pear-shaped separating funnel according to a certain ratio of O/A for a certain period of time in an air bath constant temperature oscillator at a set temperature and then settled for phase separation; then, the concentration of As^III^ in the liquid after exchange was analyzed. In this manner, the As^III^ extraction rate of ${\text{CO}}_{3}^{2-}$-type TOMAC was obtained.

#### Loaded Organic Reverse Extraction Experiment

After a stripping agent was prepared according to a certain concentration, it was placed into a pear-shaped funnel with loaded organic phase at a certain O/A ratio. Then, the phase was separated at room temperature after mixing in the KS oscillator for a certain period of time. Then, the As^III^ concentration in the solution after conversion was analyzed to obtain its stripping rate in the loaded organic phase.

### Analysis Method and Data Processing

#### Detection of As Content in Solution

The concentration of As is mainly determined using potassium bromate titration in GB/T3884-20128. Using KBr as the catalyst, arsenic (V) is reduced to a lower valence state in HCl; thereafter, arsenic is separated with As_2_Cl_3_. After the absorption of water by As_2_Cl_3_, sodium p-dimethylaminoazobenzene sulfonate is used as an indicator. Finally, As is titrated with a prepared KBrO_3_ standard solution. The final concentration of As is expressed by Equation (1),

(1) $$\begin{array}{l} \beta =V_{2}\times c_{1}\times M_{1}/V_{1}\times 10^{3}, \end{array}$$

where β is the concentration of As (mg/l), *V*_1_ is the volume of the aqueous phase (ml), *V*_2_ is the volume of KBrO_3_ consumed during the titration (ml), *c* is the concentration of the KBrO_3_ standard solution (mol/l), and M_1_ is the molar mass of 1/2 As (37.46 g/mol).

#### Determination of Cl^-^ Concentration in Solution

The concentration of Cl^−^ is determined by the molar method, and the analytical procedure is mainly referred to GB/T 15453-2008, with a determination range of 10–120 mg/l. For the pH value of 5.0–9.5, using K_2_CrO_4_ as the indicator, AgNO_3_ solution with a known accurate concentration was used to titrate the sample to be determined. AgNO_3_ reacts with chloride to produce AgCl, and excessive AgNO_3_ reacts with K_2_CrO_4_ to produce Ag_2_CrO_4_. When a precipitate is produced, the indicator stops dropping. The final concentration of Cl^−^ is expressed by Equation (2),

(2) $$\begin{array}{l} \rho =V_{4}\times c_{2}\times M_{2}/V_{3}\times 10^{3}, \end{array}$$

where ρ is the concentration of Cl^−^ (mg/l), *V*_3_ is the volume of the aqueous phase (ml), *V*_4_ is the volume of AgNO_3_ consumed during the titration (ml), *c* is the concentration of the AgNO_3_ standard solution (mol/l), and M_2_ is the molar mass of Cl^−^ (35.50 g/mol).

#### Determination of ${\text{CO}}_{3}^{2-}$ and OH^-^ Concentrations in Solution

The concentrations of ${\text{CO}}_{3}^{2-}$ and OH^−^ were determined using titration. The analysis steps were mainly referred to as DZ/T0064.49-93; the detection range for ${\text{CO}}_{3}^{2-}$ and OH^−^ concentration was no less than 10 and 4 mg/l, respectively. The titrant solution of HCl with a precise known concentration was used; the indicators were phenolphthalein and sodium dimethylaminoazobenzene sulfonic acid solution.

After the addition of an appropriate volume (*V*) of the feed solution into a conical flask, a few drops of phenolphthalein were added. If the solution turned red, HCl solution with a precise known concentration was added until the red color in the feed solution faded, and its added amount (*V*_5_) was recorded. Subsequently, three drops of sodium dimethylaminoazobenzene sulfonic acid solution was also added into the conical flask. The continuous addition of HCl solution with a precise known concentration was performed until the feed solution turned orange, and the amount of HCl (*V*_6_) added was recorded.

The contents of ${\text{CO}}_{3}^{2-}$ and OH^−^ are expressed by Equations (3) and (4), respectively:

(3) $$\begin{array}{l} C_{1}=\left(V_{5}-V_{6}\right)\times c_{3}\times M_{3}/V_{7}\times 10^{3}, \end{array}$$

(4) $$\begin{array}{l} C_{2}=2V_{6}\times c_{3}\times M_{4}/V_{7}\times 10^{3}, \end{array}$$

where *C*_1_ and *C*_2_ are the concentrations of OH^−^ and ${\text{CO}}_{3}^{2-}$ (mg/l), respectively, *V*_5_ and *V*_6_ are the volumes of HCl consumed by the first and second titrations (ml), respectively, *V*_7_ is the volume of the aqueous phase (ml), *c* is the concentration of the HCl standard solution (mol/l), and M_3_ and M_4_ are the molar mass of OH^−^ (17.01 g/mol) and 1/2 ${\text{CO}}_{3}^{2-}$ (30.01 g/mol), respectively.

#### Extraction Rate and Back Extraction Rate of As

After solvent extraction, the extraction rate of As is calculated using Equation (5),

(5) $$\begin{array}{l} \eta =\left(C_{3}\times V_{8}-C_{4}\times V_{9}\right)/\left(C_{3}\times V_{8}\right)\times 100\%. \end{array}$$

The arsenic-supported organic phase is back-extracted, whereas the back extraction rate of arsenic is calculated by Equation (6):

(6) $$\begin{array}{l} \sigma =\left(C_{5}\times V_{10}\right)/\left(C_{3}\times V_{8}-C_{4}\times V_{9}\right)\times 100\%, \end{array}$$

where η is the extraction ratio of As (%), σ is the stripping ratio of As (%), *C*_3_ is the concentration of As in the feed solution (g/l), *V*_8_ is the volume of feed solution (ml), *C*_4_ is the concentration of As in the raffinate (g/l), *V*_9_ is the volume of raffinate (ml), *C*_5_ is the concentration of As in the strip liquor (g/l), and *V*_10_ is the volume of strip liquor (ml).

## Results and Discussion

### The Transformation of TOMAC

#### ${\text{CO}}_{3}^{2-}$ Transformation Mechanism of TOMAC

According to the literature (Wu et al., 2017; Buev et al., 2018), the more lipophilic anions are more likely to react with the quaternary ammonium cations and enter the organic phase. The order of association of quaternary ammonium cations with each anion is roughly as follows:

$$\begin{array}{l} ClO_{4}^{-}>I^{-}>>ClO_{3}^{-}>Cl^{-}>HSO_{4}^{-}>HCO_{3}^{-}>SO_{4}^{2-}\\ \;>OH^{-}>CO_{3}^{2-}>S^{2-}>AsO_{4}^{3-}>SiO_{3}^{2-} \end{array}$$

From the above, the association ability of Cl^−^ to quaternary ammonium salt cation is evidently relatively strong, whereas that of arsenic anion is relatively weak, which is the main reason for the inhibition of arsenic extraction. A larger anion radius corresponds to a smaller charge, and a lower degree of hydration of the aqueous solution corresponds to a greater advantage in extraction (Suflet et al., 2015; Chauhan and Kaur, 2017). It can be inferred that the association ability of ${\text{HAsOS}}_{2}^{2-}$ is between ${\text{CO}}_{3}^{2-}$ and ${\text{AsO}}_{4}^{3-}$, and the extraction ability of arsenic may be improved if the Cl^−^ quaternary ammonium salt is transformed into the ${\text{CO}}_{3}^{2-}$ type. However, the direct transformation process may not be easy to perform, because the strong association anion is easy to exchange with the weak association anion; otherwise, it is very difficult.

The experimental results of direct transformation of TOMAC by 8% Na_2_CO_3_ showed that under the conditions of 30% TOMAC + 15% sec-octyl alcohol + 55% sulfonated kerosene, V_O_/V_A_ = 1/1, the compositions of water phase are 0.5 mol/l NaOH, the concentration of As^III^ is 9.69 × 10^−2^ mol/l, and as the transformation times range from 1 to 5, the extraction rate of As^III^ is from 47.3 to 63.7%. The extraction rate hardly increased with the transformation time increase. In view of the difficulty of the direct conversion from Cl^−^-type TOMAC to ${\text{CO}}_{3}^{2-}$-type TOMAC, this study also considered a step-by-step conversion method. Because the associative ability of ${\text{HCO}}_{3}^{-}$ is clearly stronger than that of ${\text{CO}}_{3}^{2-}$ and weaker than that of Cl^−^, based on the principle of concentration gradient equilibrium, the Cl^−^-type quaternary ammonium salt is first converted into ${\text{HCO}}_{3}^{-}$-type by a high-concentration ${\text{HCO}}_{3}^{-}$ solution. Then, the final conversion of ${\text{CO}}_{3}^{2-}$ is achieved by alkaline washing.

The conversion process of Cl^−^- to ${\text{HCO}}_{3}^{-}$-type TOMAC can be expressed by the following Equation (7):

(7) $$\begin{array}{l} R_{4}NCl+HCO_{3}^{-}\to R_{4}NHCO_{3}+Cl^{-}. \end{array}$$

The intermediate product ${\text{HCO}}_{3}^{-}$-type TOMAC is subjected to alkaline washing, and the reaction expressed in Equation (8) is

(8) $$\begin{array}{l} 2R_{4}NHCO_{3}+2OH^{-}\to {\left(R_{4}N\right)}_{2}CO_{3}+CO_{3}^{2-}+2H_{2}O. \end{array}$$

#### ${\text{CO}}_{3}^{2-}$ Transformation Process of TOMAC

**Figure 3:**
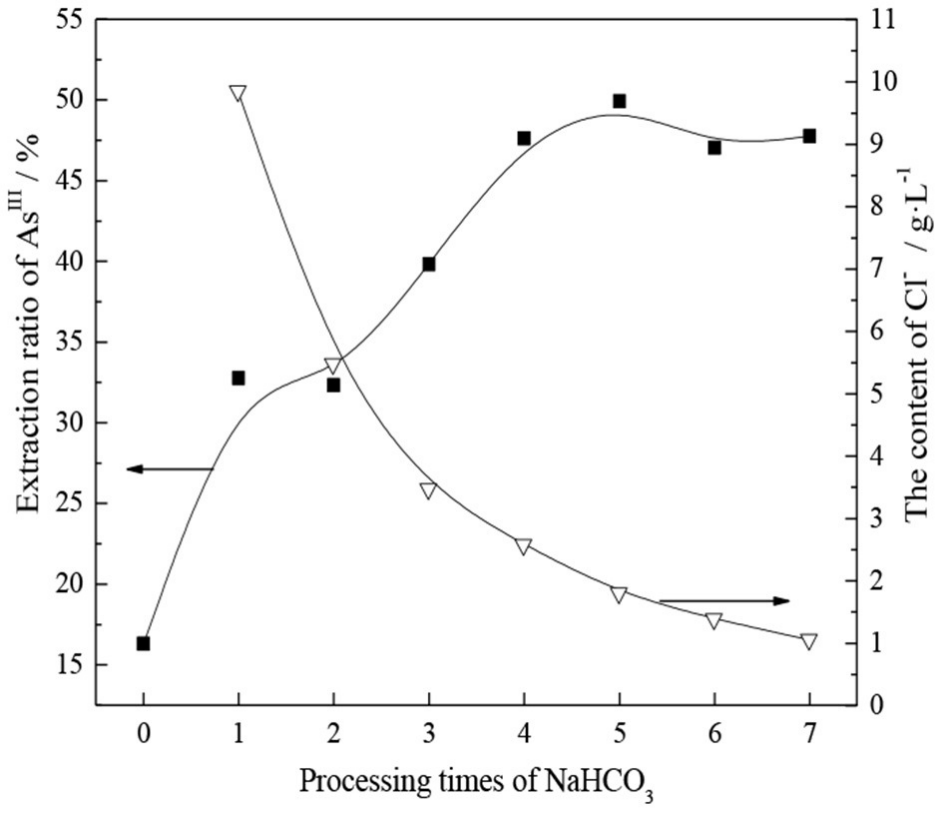
Relationship between the extraction rate of As^III^ and the number of treatments with saturated NaHCO_3_.

The experiment was conducted using a saturated NaHCO_3_ aqueous solution as a transition agent, and the effect of the number of treatments with saturated NaHCO_3_ solution on the conversion of TOMAC from Cl^−^- to ${\text{HCO}}_{3}^{-}$-type was investigated. The experimental results are shown in Figure 3.

As shown in Figure 3, the extraction rate of As^III^ from TOMAC significantly increases after the saturated NaHCO_3_ treatment, from 16.3 to 32.8% after one saturated NaHCO_3_ treatment. This shows that improving the extraction capacity of As^III^ is feasible through the transformation of TOMAC. Further, Figure 3 shows that with increasing number of times of contact between organic phase and saturated NaHCO_3_ solution, the concentration of Cl^−^ in the solution after conversion successively decreases, indicating that the transformation efficiency is constantly improving. Moreover, as the concentration of Cl^−^ in the solution after conversion decreases and gradually reaches equilibrium, the extraction rate of As^III^ after the extractant transformation continues to increase to 47.5%, until a significant change is no longer observed. In summary, after treating the organic phase with saturated NaHCO_3_ five times, the Cl^−^-type quaternary ammonium salt can be effectively converted into ${\text{HCO}}_{3}^{-}$-type quaternary ammonium salt.

**Figure 4:**
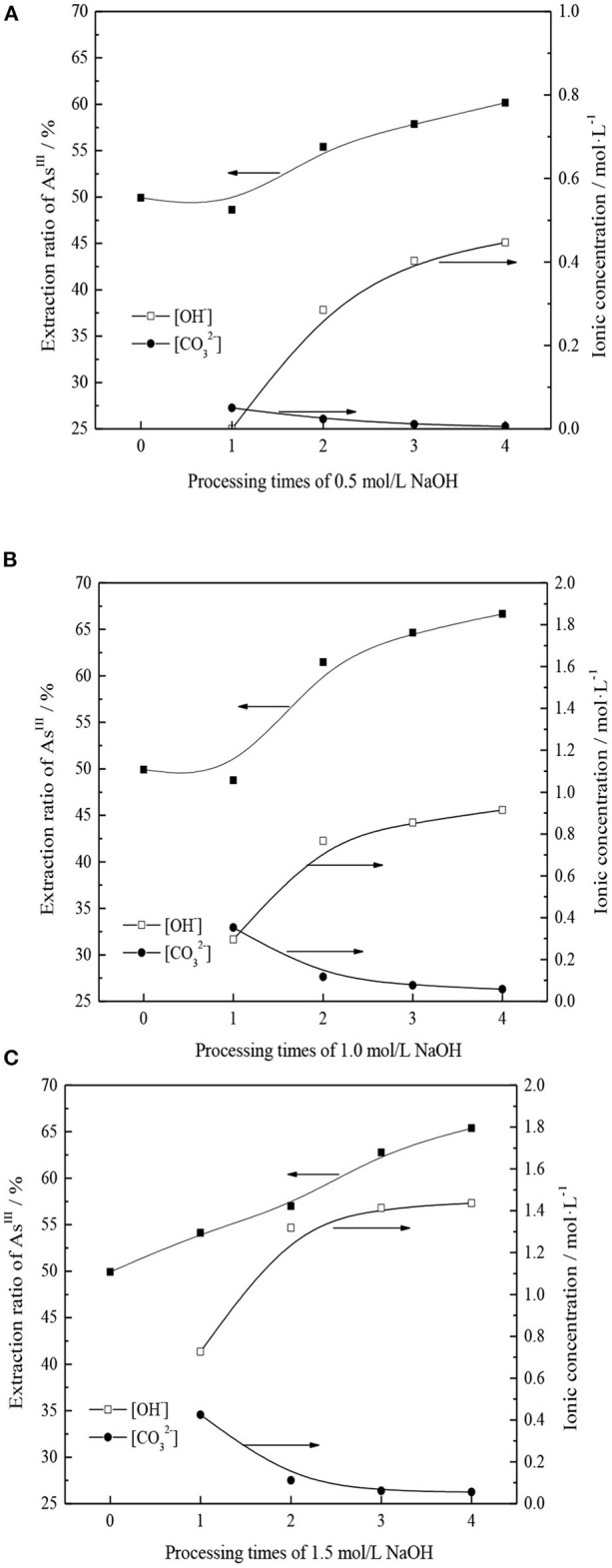
**(A)** NaOH concentration is 0.5 mol/L and treatment times on As^III^ extraction rate and concentration of ions in alkaline washing solution. **(B)** NaOH concentration is 1.0 mol/L and treatment times on As^III^ extraction rate and concentration of ions in alkaline washing solution. **(C)** NaOH concentration is 1.5 mol/L and treatment times on As^III^ extraction rate and concentration of ions in alkaline washing solution.

From section ${\text{CO}}_{3}^{2-}$ Transformation Mechanism of TOMAC, TOMAC of ${\text{HCO}}_{3}^{-}$ type can be further transformed into ${\text{CO}}_{3}^{2-}$ type by alkali washing. However, in the process of alkali washing, if the concentration of NaOH is too high, TOMAC may be transformed into R_4_NOH, with poor stability. If the concentration of NaOH is too low, the number of alkali washings may need to increase, which affects the transformation efficiency (Weisshaar et al., 2012). Therefore, the effects of NaOH concentration and the number of alkali washings on the further conversion of ${\text{HCO}}_{3}^{-}$-type quaternary ammonium salt and the extraction rate of As^III^ were investigated, respectively, and the experimental results are shown in Figure 4.

As shown in Figure 4, with the NaOH concentration range of 0.5–1.0 mol/l in the wash solution, the treatment has little effect on the As^III^ extraction rate; however, the As^III^ extraction rate increases more significantly with an increase in the number of treatments. For an NaOH concentration of 1.5 mol/l, the extraction rate of As^III^ increases with an increase in the number of treatments. The extraction efficiency of As^III^ increases with the number of alkali washings owing to the transformation of TOMAC from ${\text{HCO}}_{3}^{-}$ to ${\text{CO}}_{3}^{2-}$ type.

**Figure 5:**
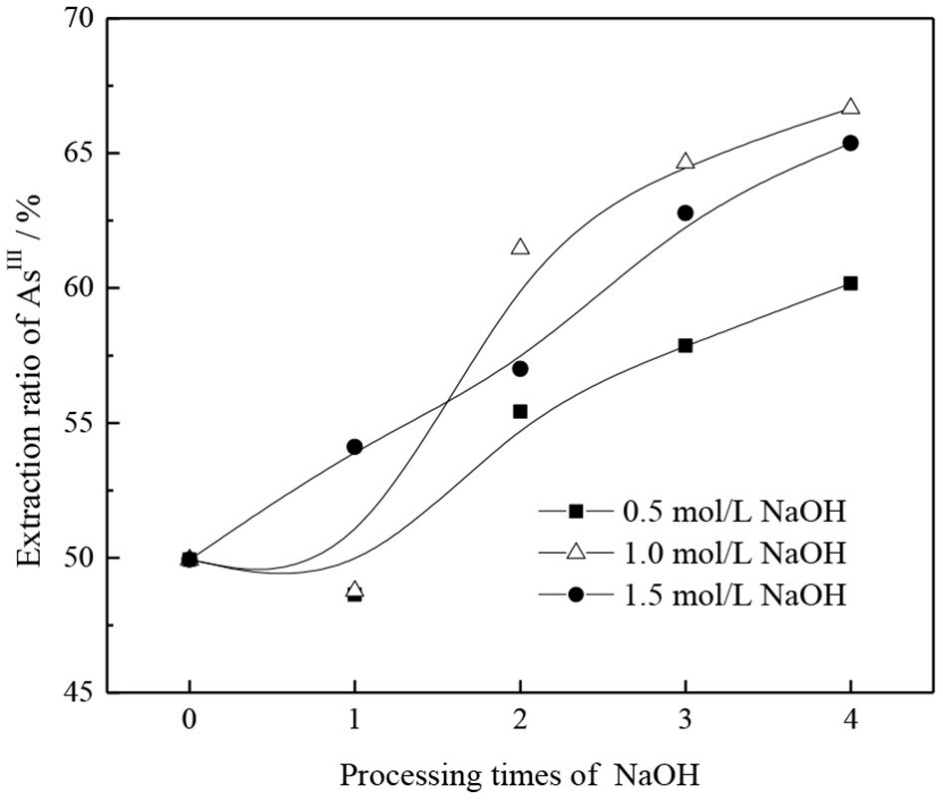
Effect of alkaline washing times on As^III^ extraction rate under different NaOH concentrations.

The effects of NaOH concentration and number of alkaline washings on the As^III^ extraction rate were comprehensively analyzed, and results are shown in Figure 5. Under different alkali concentration conditions, with an increase in the number of alkali washings, the pattern of successive increases of As^III^ extraction rate remains basically the same. With an increase in the NaOH concentration from 0.5 to 1.0 mol/l, the promotion effect of alkaline washing on As^III^ extraction can be clearly demonstrated only when the number of alkaline washings is more than two. When the NaOH concentration is further increased to 1.5 mol/l and the number of alkaline washings is more than two, the extraction rate of As^III^ is significantly better than that obtained at 0.5 mol/l NaOH but lower than that obtained at 1.0 mol/l NaOH. This may be due to the fact that with an increase in alkalinity, in addition to the neutralization reaction of ${\text{HCO}}_{3}^{-}$ group in organic phase, excessive OH^−^ replaces the generated ${\text{CO}}_{3}^{2-}$ groups. Owing to the relatively stronger OH^−^ association ability, the As^III^ extraction capacity is reduced.

Based on the above, the extraction rate of As^III^, the amount of NaOH, and the conversion efficiency are comprehensively considered to convert TOMAC from ${\text{HCO}}_{3}^{-}$ to ${\text{CO}}_{3}^{2-}$ type effectively; 1.0 mol/l NaOH solution was selected for washing three times.

It can be known from the above experimental results and analysis that ${\text{CO}}_{3}^{2-}$-type TOMAC has a significant effect on the extraction rate of As^III^ in alkaline solution. The transformation process of TOMAC is accomplished in two steps, Cl^−^ to ${\text{HCO}}_{3}^{-}$ type and ${\text{HCO}}_{3}^{-}$ to ${\text{CO}}_{3}^{2-}$ type; how to improve the transformation rate of these two processes is the critical process.

#### Infrared Spectrum Analysis Before and After ${\text{CO}}_{3}^{2-}$ Transformation of TOMAC

**Figure 6:**
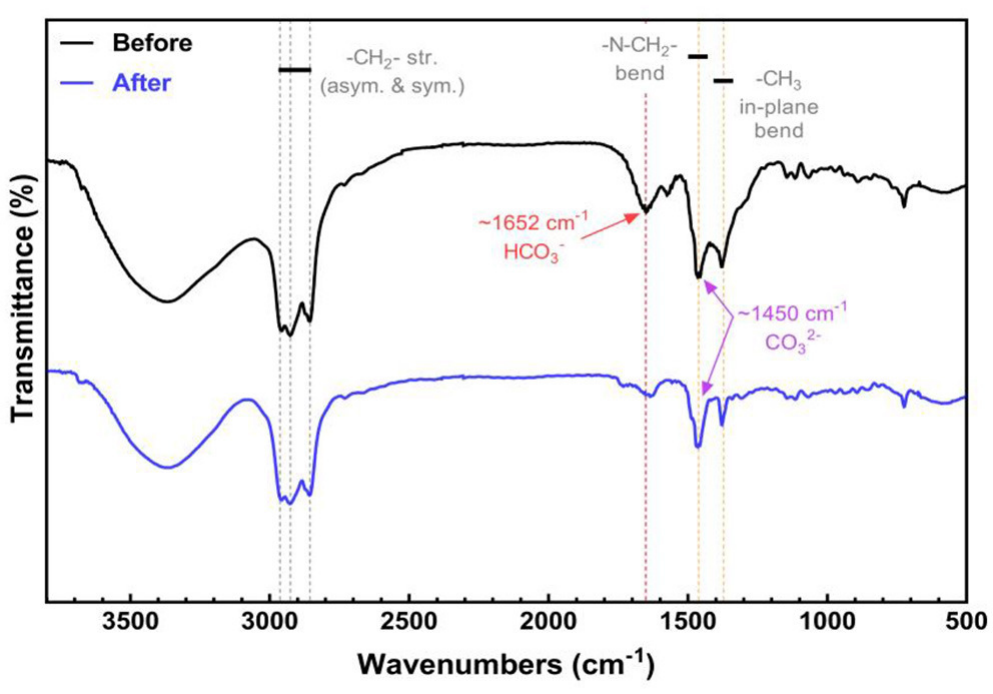
Infrared spectra of TOMAC before and after transformation.

TOMAC before and after transformation (${\text{CO}}_{3}^{2-}$ type) is detected and analyzed, respectively, by Fourier transform infrared spectroscopy, and the obtained infrared spectrum is shown in Figure 6.

Figure 6 represents the FTIR spectra of TOMAC before and after the transformation. The peaks at ~2,963, ~2,926, and ~2,856 cm^−1^ can be assigned to the -CH_2_- stretching. The characteristic vibration bands at ~1,462 and ~1,372 cm^−1^ represent the N-CH_2_- bending and in-plane bending of -CH_3_, respectively. The characteristic IR band of ${\text{HCO}}_{3}^{-}$ at ~1,652 cm^−1^ can be found in the spectrum of TOMAC before the transformation. However, the characteristic peak of ${\text{HCO}}_{3}^{-}$ disappears after the NaOH treatment, revealing the transformation from ${\text{HCO}}_{3}^{-}$ to ${\text{CO}}_{3}^{2-}$. It should be noted that the characteristic peak of ${\text{CO}}_{3}^{2-}$ locates at ~1,450 cm^−1^, which overlaps with the vibration band region of N-CH_2_- bending (Larkin, 2011). Thus, it is difficult to distinguish the two vibration bands from the FTIR spectra.

### Extraction of Thioarsenious Acid by ${\text{CO}}_{3}^{2-}$-Type TOMAC

#### Extraction Mechanism of ${\text{CO}}_{3}^{2-}$-Type TOMAC

The extraction capacity of thioarsenious acid in the ${\text{CO}}_{3}^{2-}$-type TOMAC organic phase was determined by the continuous saturation method (Zhu, 2005).

**Figure 7:**
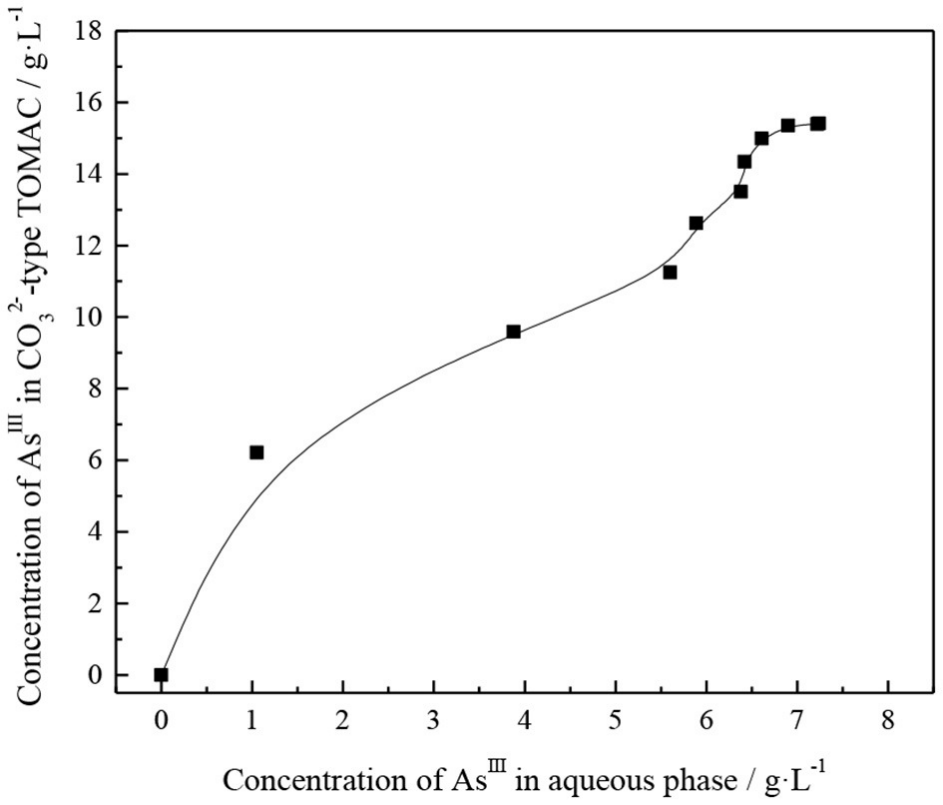
Isotherm of thioarsenite extraction.

First, the organic phase of 30% TOMAC + 15% sec-octyl alcohol + 55% sulfonated kerosene was transformed into ${\text{CO}}_{3}^{2-}$, and the aqueous solution containing arsenic was presulfurized. After the transformation, the organic phase was repeatedly contacted with fresh water phase under the condition of phase ratio O/A = 1/1 and vibrated and mixed for 10 min each time. The concentration of As^III^ in the raffinate was analyzed after each extraction reached equilibrium; then, the content of As^III^ in the organic phase was calculated by subtraction method and accumulated step by step. The concentration of As^III^ in the raffinate is basically the same as that in the aqueous solution before extraction, until no significant change is observed in the As^III^ concentration in the loaded organic phase. The extraction isotherms were plotted based on the concentration relationship between As^III^ in the loaded organic phase and that in the equilibrium aqueous phase, as shown in Figure 7.

In Figure 7, the As^III^ content enriched in the ${\text{CO}}_{3}^{2-}$-type TOMAC increases with increasing number of times of contact between the ${\text{CO}}_{3}^{2-}$-type TOMAC and the feed liquid. The content of As^III^ in raffinate was almost the same as that in the feed after ${\text{CO}}_{3}^{2-}$-type TOMAC was contacted with the feed nine times; thus, the organic phase can be considered to have been saturated, and the saturation capacity of ${\text{CO}}_{3}^{2-}$-type TOMAC for extracting thioarsenious acid was 15.41 G/l (0.21 mol/l). When ${\text{CO}}_{3}^{2-}$-type TOMAC is used to extract thioarsenious acid, the following reactions occur:

(9) $$\begin{array}{l} {\left(R_{4}N\right)}_{2}CO_{3}+HAsOS_{2}^{2-}\to {\left(R_{4}N\right)}_{2}HAsOS_{2}+CO_{3}^{2-}. \end{array}$$

(10) $$\begin{array}{l} {\left(R_{4}N\right)}_{2}CO_{3}+HAsO_{2}S^{2-}\to {\left(R_{4}N\right)}_{2}HAsO_{2}S+CO_{3}^{2-}. \end{array}$$

#### Effect of Volume Fraction of ${\text{CO}}_{3}^{2-}$-Type TOMAC on As^III^ Extraction

**Figure 8:**
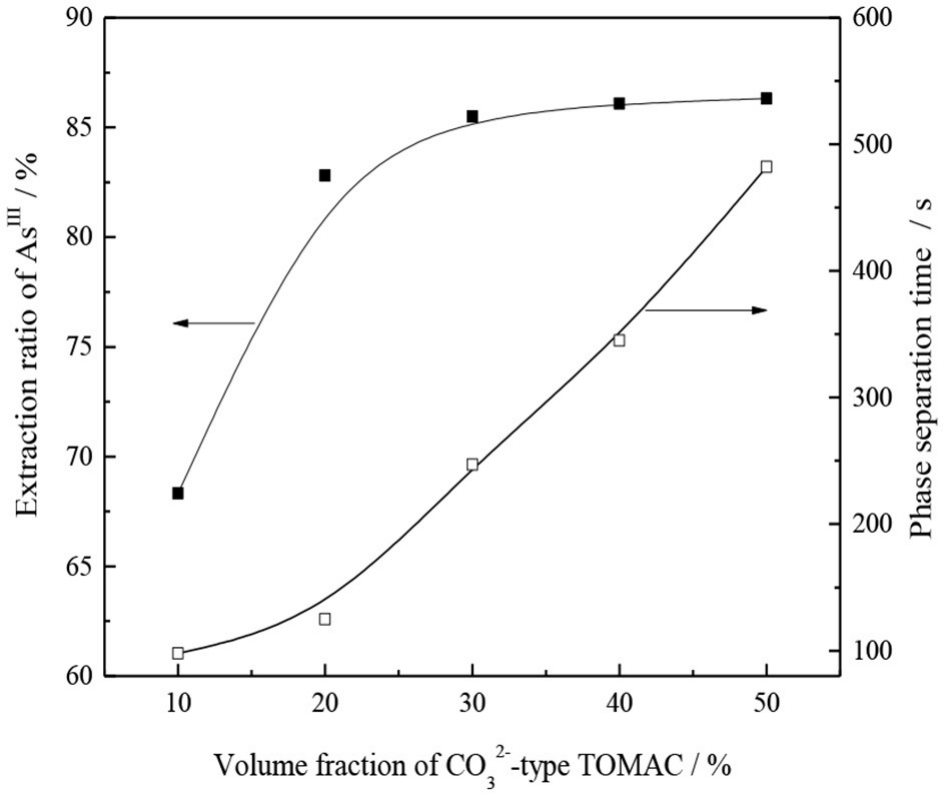
Effect of volume fraction of ${\text{CO}}_{3}^{2-}$-type TOMAC on As^III^ extraction and phase separation.

Under the conditions of 15% secondary octanol concentration, V_O_/V_A_ = 1/1, and normal temperature, oil, and water were mixed and shaken for 10 min. Then, the effects of the volume fraction of ${\text{CO}}_{3}^{2-}$-type TOMAC on the single-stage extraction rate of As^III^ and oil–water phase separation were investigated, and the experimental results are shown in Figure 8.

As seen from Figure 8, for a TOMAC concentration within 30%, the As^III^ extraction rate has a significant increase trend with an increase in the volume fraction of ${\text{CO}}_{3}^{2-}$-type TOMAC. As the volume fraction of ${\text{CO}}_{3}^{2-}$-type TOMAC increases from 10 to 30%, the extraction yield of As^III^ increases from 68.3 to 85.5% and further increases to 50% with the volume fraction of the ${\text{CO}}_{3}^{2-}$-type TOMAC. The extraction rate of As^III^ slightly increases to 86.3%, and the volume fraction has no significant effect on the extraction rate. As is further apparent from Figure 8, the oil–water phase time increases with the extractant concentration. When the volume fraction of ${\text{CO}}_{3}^{2-}$-type TOMAC reached 50%, the time of the oil–water phase was prolonged to nearly 9 min, which was unfavorable to the extraction operation.

The volume fraction of ${\text{CO}}_{3}^{2-}$-type TOMAC was 30%, the extraction yield of As^III^ was 85.5%, and the time of the phase separation was ~4 min.

#### Effect of Volume Fraction of 2-octanol

**Figure 9:**
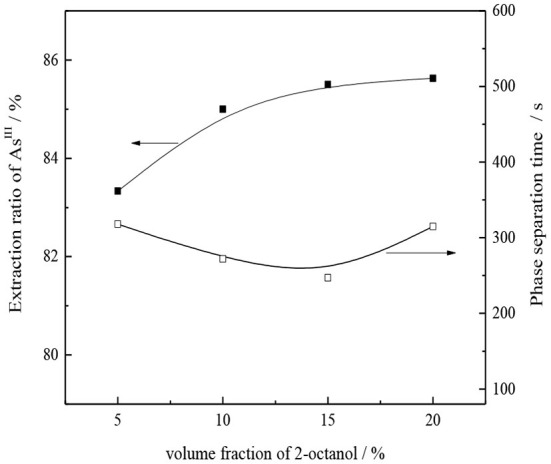
Effect of volume fraction of 2-octanol on As^III^ extraction and phase separation.

The use of DL-2-octanol as the polarity improver is conducive to the depolymerization of ${\text{CO}}_{3}^{2-}$-type TOMAC (Uslu, 2008), thereby increasing the effective ion concentration of the extractant and improving the extraction effect. Under the conditions of 30% ${\text{CO}}_{3}^{2-}$-type TOMAC (volume fraction), V_O_/V_A_ = 1/1, and room temperature, the effect of DL-2-octanol concentration on the As^III^ single-stage extraction rate and oil–water phase separation was investigated by oscillating the oil–water mixture for 10 min, and results are shown in Figure 9.

As seen from Figure 9, as the secondary octanol concentration increases from 5 to 15%, the As^III^ extraction yield slightly increases from 83.3 to 85.5%. With the further increase in the amount of 2-octanol, the extraction of As^III^ shows no significant improvement; however, the loss of organic phase in water may increase. Therefore, a suitable concentration of 2-octanol is 15%, for an oil–water phase time of ~4 min, in which the phase separation is relatively fast and clear.

#### Effect of Extraction Temperature

**Figure 10:**
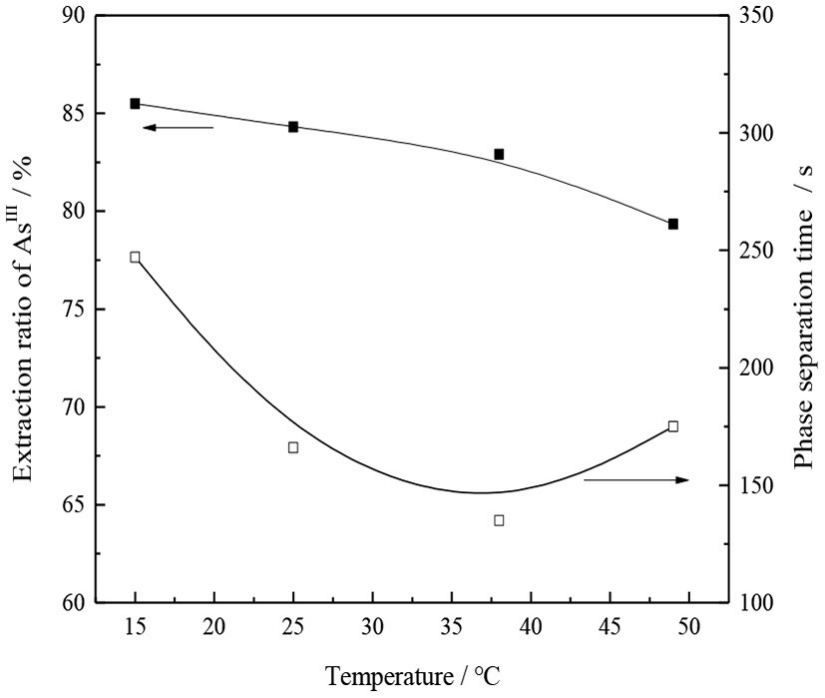
Effect of temperature on As^III^ extraction and phase separation.

The transfer rate between the oil and water phases can be affected by the change in temperature, which may also affect the equilibrium of extraction reaction. Under the condition of 30% ${\text{CO}}_{3}^{2-}$-type TOMAC + 15% secondary octanol + 55% sulfonated kerosene and V_O_/V_A_ = 1/1, the oil and water were mixed and oscillated for 10 min, and the effect of temperature change on the single-stage extraction rate of As^III^ was investigated while the oil and water phases were carried out at room temperature. The experimental results are shown in Figure 10.

With the increase in the temperature, the viscosity of the organic phase normally decreases, which facilitates the extraction process; however, for the TOMAC extraction of As^III^, the As^III^ extraction rate does not increase but rather decreases with an increase in the extraction temperature (see Figure 10).

According to van't Hoff equation of the chemical reaction (Richards, 1926; Deiters, 2012),

(11) $$\begin{array}{l} \frac{\text{d}\ln\;K}{dT}=\frac{\Delta H}{RT^{2}}, \end{array}$$

where *K* is the equilibrium constant, *T* is the absolute temperature (°C), Δ*H* is the enthalpy change (kJ/mol), and *R* is the universal gas constant (8.314 J·mol^−1^·K^−1^).

As Δ*H* does not vary significantly with temperature and the temperature range discussed in this experiment is narrow, Δ*H* can be assumed to have a certain value, independent of temperature. Integrating Equation (11), we get

(12) $$\begin{array}{l} \ln\;K=-\frac{\Delta H}{RT}+C\left(Cisconstant\right). \end{array}$$

Equation (11) shows that the equilibrium constant of the reaction is linear with 1/*T*.

As most of the thioarsenious acid in the system exists in the form of ${\text{HAsOS}}_{2}^{2-}$, to simplify the calculation, only the reaction in Equation (9) is considered, from which the equilibrium constant *K*_*ex*_ is derived,

(13) $$K_{ex}=\frac{\left[{\left({\text{R}}_{4}\text{N}\right)}_{2}{\text{HAsOS}}_{2}\right]\left[{\text{CO}}_{3}^{2-}\right]}{\left[{\left({\text{R}}_{4}\text{N}\right)}_{2}CO_{3}\right]\left[{\text{HAsOS}}_{2}^{2-}\right]}.$$

According to the distribution ratio formula,

$D\text{=}\frac{\left[{\left(R_{4}N\right)}_{2}HAsOS_{2}\right]}{\left[HAsO{S_{2}}^{2-}\right]}$. (13)

From Equations (12) and (13), the relationship between the reaction equilibrium constant and distribution ratio can be obtained

(14) $$\begin{array}{l} K_{ex}=\frac{D\left[C{O_{3}}^{2-}\right]}{\left[{\left(R_{4}N\right)}_{2}C{O_{3}}^{2-}\right]}. \end{array}$$

Taking the logarithm of both sides at the same time yields

(15) $$\begin{array}{l} \ln\;K_{ex}=\ln\;D+ln\left[CO_{3}^{2-}\right]-ln{\left[{\left(R_{4}N\right)}_{2}CO_{3}\right]}^{2}. \end{array}$$

As the concentration of the extractant TOMAC is constant at 30% and the concentration of the ${\text{CO}}_{3}^{2-}$ ion changes slightly, the last two terms in Equation (15) can be assumed to be constants for the sake of simplification. Consequently,

(16) $$\begin{array}{l} \ln\;D=-\frac{\Delta H}{RT}+C^{\prime }\;\left(C^{\prime }isconstant\right). \end{array}$$

Based on the experimental data, the extraction rate, distribution ratio, and other parameters obtained under different temperature conditions are calculated, and the results are shown in Table 1.

**Table 1:** Parameters such as extraction rate and partition ratio at different temperatures.

**Temperature (°>C)**	**Extraction rate (%)**	**Distribute ratio *D***	**ln*D***	**1/*T* (K^**−1**^)**
15	85.5	5.91	1.78	0.00347
25	84.30	5.37	1.68	0.00336
38	82.09	4.58	1.52	0.00322
49	79.34	3.84	1.35	0.00311

**Figure 11:**
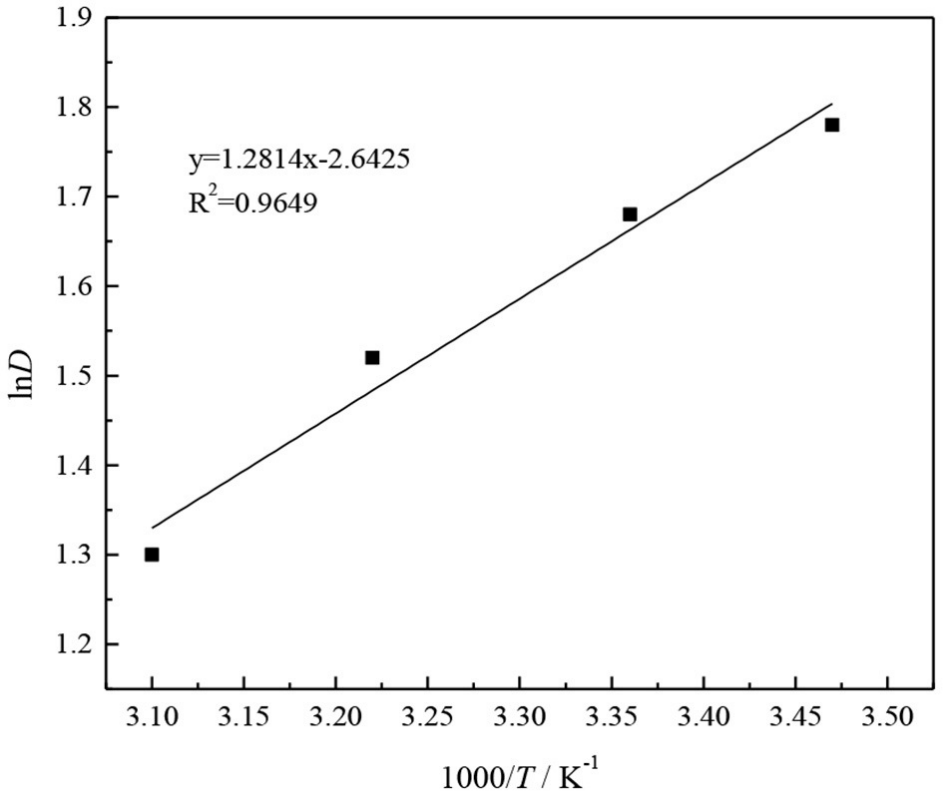
Relationship between 1/*T* and ln*D* and fitting results.

Plot and fit 1/*T* and ln*D* in Table 1, and the result is shown in Figure 11.

As can be seen from Figure 11, 1/*T* and ln*D* have a linear relationship, and the fitted equation is

(17) $$\begin{array}{l} y=1.2814x-2.6425\left(R^{2}=0.9649\right). \end{array}$$

From Equation (17), we can see that the slope of the fitted straight line is 1.2814.

(18) $$\begin{array}{l} \Delta H=-R\times 1.2814=-10.6536kJ/mol. \end{array}$$

From the calculation, it is evident that the process of extracting thioarsenious acid with ${\text{CO}}_{3}^{2-}$-type TOMAC is an exothermic reaction, and when the extraction temperature increases, the equilibrium of the extraction reaction proceeds in the reverse direction, which is not conducive to the extraction of As^III^.

As seen from Figure 10, as the extraction temperature increases from 15 to 50°C, the extraction ratio of As^III^ decreases from 85.5 to 79.33%, which indicates that the extraction of thioarsenious acid by ${\text{CO}}_{3}^{2-}$-type TOMAC is an exothermic reaction. Although the temperature increase speeds up the phase separation, it is still not conducive to the extraction of As^III^; thus, with comprehensive consideration, the extraction process should be conducted at room temperature.

#### Effect of Extraction Time

**Figure 12:**
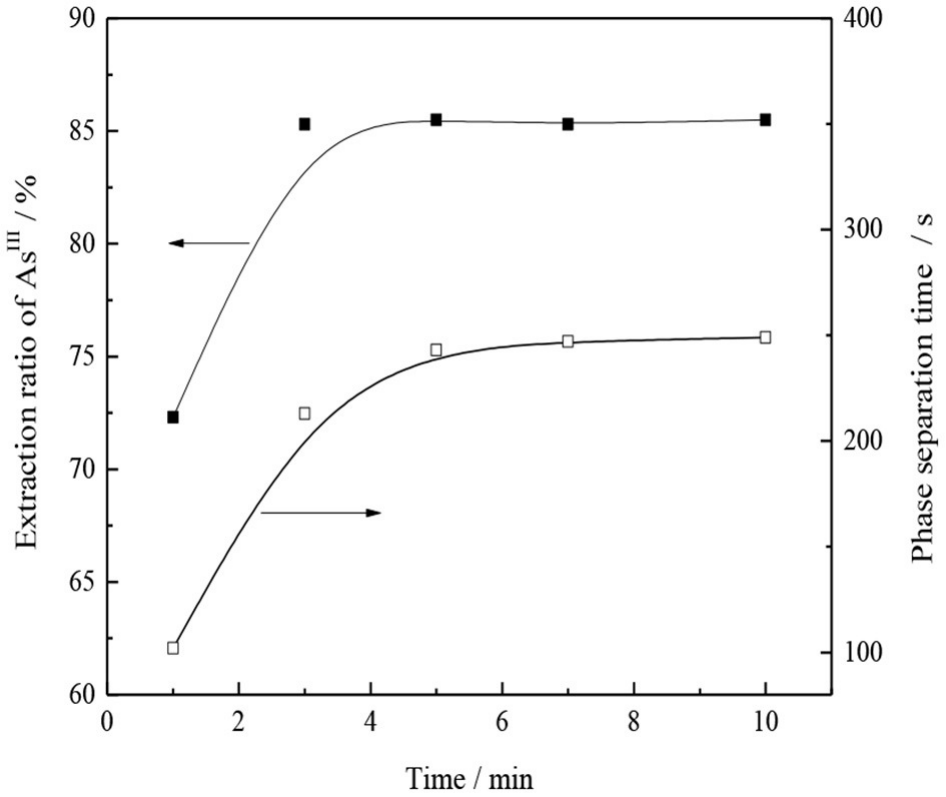
Effect of extraction time on As^III^ extraction and phase separation.

Under the conditions of organic phase composition 30% ${\text{CO}}_{3}^{2-}$-type TOMAC + 15% secondary octanol + 55% sulfonated kerosene, V_O_/V_A_ = 1/1, and normal temperature, the effects of the oil–water mixed extraction time on the single-stage extraction rate of As^III^ and oil–water phase separation are investigated, and the experimental results are shown in Figure 12.

As shown in Figure 12, the extraction of As^III^ can reach equilibrium in ~3 min, extraction rate of As^III^ reaches 85.2%, and extraction rate of As^III^ does not change significantly with the further extension of the extraction time, which indicates that the extraction process is relatively fast and the phase separation is also fast, i.e., ~4 min. To ensure the full mixing of oil and water, an extraction time of 5 min was selected under the experimental conditions.

#### Effect of Phase Ratio

**Figure 13:**
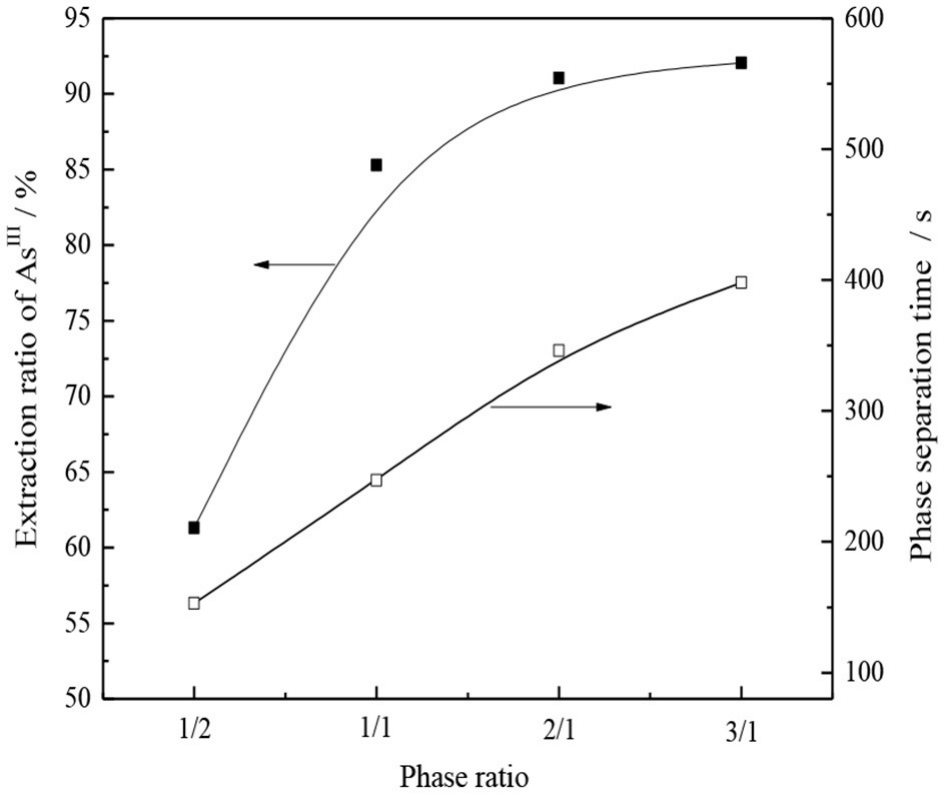
Effect of phase ratio on As^III^ extraction and phase separation.

Under the condition of organic phase composition 30% ${\text{CO}}_{3}^{2-}$-type TOMAC + 15% secondary octanol + 55% sulfonated kerosene and extraction for 5 min at room temperature, the effect of the oil–water contact ratio on the single-stage extraction rate and phase separation of As^III^ was investigated, and the experimental results are shown in Figure 13.

As seen from Figure 13, the extraction rate of As^III^ and the phase separation time increase with an increase in the phase ratio. When the V_O_/V_A_ ratio increased from 1/1 to 2/1, the extraction yield of As^III^ increased from 85.2 to 91.0%, and the time of the phase separation was prolonged from 4 to nearly 6 min. When the ratio of V_O_/V_A_ increased further to 3/1, the increase in the extraction yield of As^III^ was not significant; however, the time of the phase separation was prolonged to nearly 7 min, which was disadvantageous to the extraction process. Therefore, relatively speaking, V_O_/V_A_ = 1/1 was a better choice.

### Countercurrent Extraction Experiment

Based on the single-stage extraction experiment, the simulated countercurrent cascade extraction experiment was conducted. Based on the Kremser–Brown–Souders equation (Szitkai et al., 2002; Ushenoy and Fraser, 2003), as shown in Equation (19), the theoretical extraction stage is calculated and determined by means of the distribution ratio, phase ratio, extraction ratio, and set value of the arsenic content in the raffinate.

(19) $$\begin{array}{l} \varphi_{M}\text{=}\{\begin{array}{c} \frac{E_{M}-1}{E_{M}^{N+1}-1}\\ \frac{1}{N+1},E=1 \end{array}\text{,}E\neq 1, \end{array}$$

where ϕ_*M*_ is the fraction extracted from *M* components, *E*_*M*_ is the extraction ratio of *M* components (°C), and *N* is the theoretical series.

When the ratio O/A is 1/1, the distribution ratio *D*_*As*_ of the As^III^ extraction is calculated from Equation (13) as follows:

(20) $$\begin{array}{l} D_{As}=\frac{6.19}{1.07}=5.79. \end{array}$$

Thus, the extraction ratio *E*_*As*_ is

(21) $$\begin{array}{l} E_{As}=D_{As}\times ratio=5.79. \end{array}$$

After the cascade countercurrent extraction, the extraction rate of As^III^ is more than 99%, and the content of As^III^ in the raffinate is as low as 0.05 g/l. Thus, the raffinate fraction ϕ_As_ is

(22) $$\begin{array}{l} {\;\varphi \;}_{\text{As}}\text{=}\frac{\text{A}{\text{s}}^{\text{III}}\text{content of the raffinate liquid}}{\text{A}{\text{s}}^{\text{III}}\text{content of liquid}}\text{=}\frac{0.05}{7.26}=6.89\times 10^{-3}. \end{array}$$

In conjunction with Equations (19)–(22), the theoretical stage *N* of the extraction can be calculated:

(23) $$\begin{array}{l} N=\frac{\mathrm{lg}\left(\frac{E_{A\text{s}}-1}{\phi_{As}}+1\right)}{\mathrm{lg}E_{A\text{s}}}-1=2.73\approx 3. \end{array}$$

Therefore, the four-stage countercurrent cascade extraction can be used with the consideration of stage efficiency and other factors.

**Figure 14:**
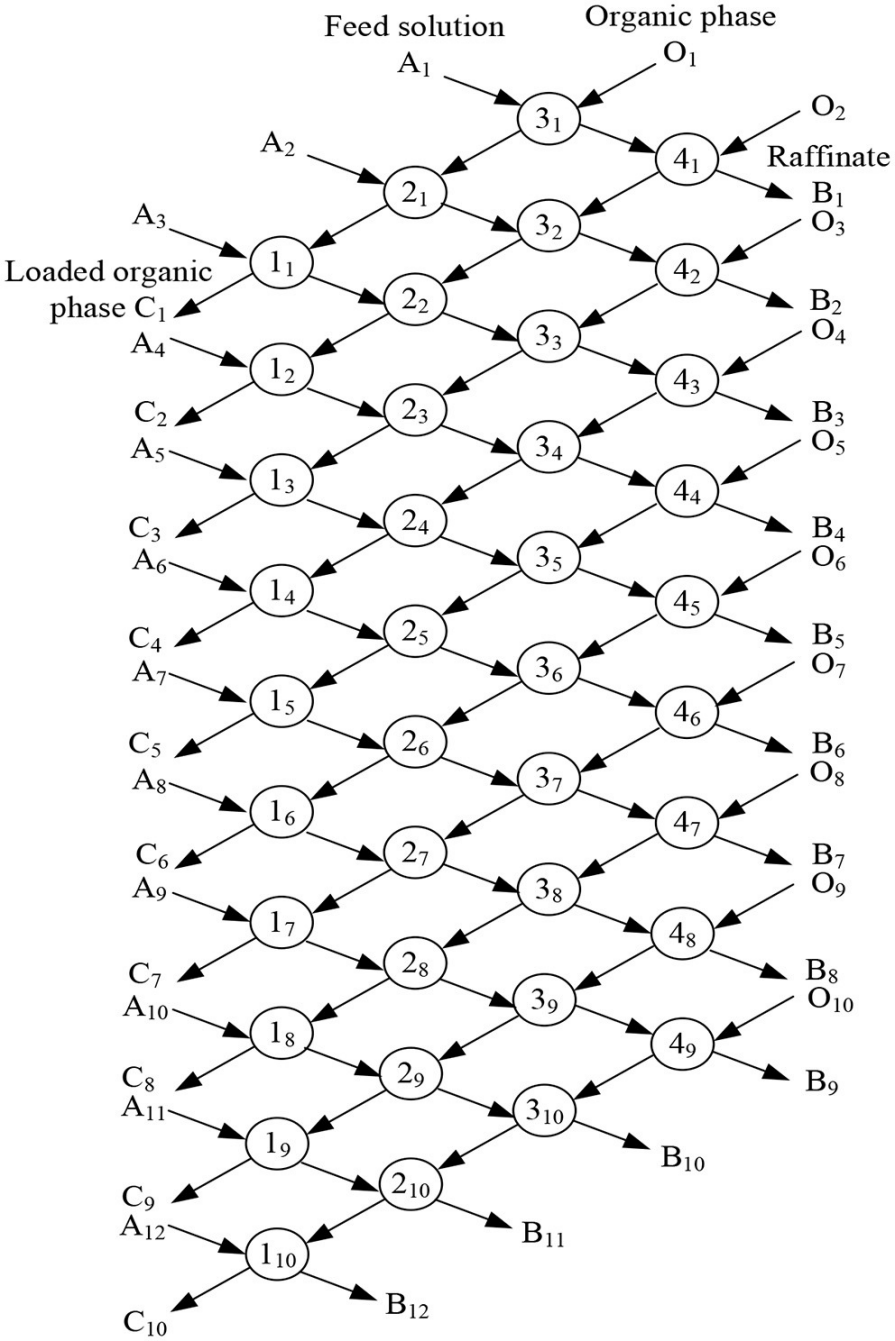
Flow chart of four-stage countercurrent extraction.

Under the condition of 30% ${\text{CO}}_{3}^{2-}$-type TOMAC + 15% sec-octyl alcohol + 55% sulfonated kerosene in the organic phase, V_O_/V_A_ = 1/1, and 5 min extraction at room temperature, the feed solution with [As^III^] = 9.69 × 10^−2^ mol/l and [NaOH] = 0.5 mol/l was extracted by four-stage countercurrent extraction. The four-stage countercurrent extraction process is shown in Figure 14.

According to the cascade countercurrent extraction theory, the solvent extraction process can be considered to be stable only when the concentration of the extracted components tends to be stable and does not change, through the analysis of the numerous extractions in the extraction process. However, it is obviously difficult to achieve this under laboratory conditions (Ma, 2009). Practical experience shows that in the cascade countercurrent extraction experiment, when the number of rows is ~3*N* – 2 times of the number of stages, the extraction process will be close to equilibrium and reach a stable concentration value (Ruiz et al., 1986). Therefore, under the laboratory conditions, a total of 10 rows were carried out in the cascade extraction simulation experiment, and the raffinate of the 8, 9, and 10th rows was analyzed for As^III^ content, with the results shown in Table 2.

**Table 2:** Experimental results of four-stage countercurrent extraction.

**Number of rows**	**Residual liquid concentration of As^**III**^ (mol·l^**-1**^)**	**Four-stage countercurrent extraction rate (%)**
8	1.33 × 10^−3^	98.62
9	1.01 × 10^−3^	98.95
10	1.31 × 10^−3^	98.64

As can be seen from Table 2, the results of the As^III^ concentration of the raffinate in rows 8, 9, and 10 are 1.34 × 10^−3^, 1.01 × 10^−3^, and 1.32 × 10^−3^ mol/l, respectively, exponential homogeneity, and it can be considered that the cascade extraction process reaches a steady state. Under the experimental conditions, the concentration of As^III^ in raffinate was reduced to less than 1.33 × 10^−3^ mol/l after the four-stage countercurrent extraction, and the extraction rate of As^III^ was more than 98.4%. The aim of removing arsenic from alkaline medium was achieved. Therefore, the extraction of thioarsenious acid with ${\text{CO}}_{3}^{2-}$-type TOMAC is an alternative method for arsenic removal from the alkaline leaching solution of metallurgical dust.

## Conclusions

In order to improve the extraction capacity for arsenic from a high-alkali solution, TOMAC is used as an extractant. The paper proposed a ${\text{CO}}_{3}^{2-}$-type tri-n-octylmethyl-ammonium chloride (TOMAC) method for extracting thioarsenite. TOMAC transformation and organic phase saturated extraction capacity were measured, and the extraction mechanism was preliminarily studied. The relationship between the extraction rate of As^III^ and the number of treatments with saturated NaHCO_3_ was investigated. The ${\text{CO}}_{3}^{2-}$ transformation process of TOMAC and infrared spectrum analysis before and after ${\text{CO}}_{3}^{2-}$ transformation of TOMAC were also studied. The results show that after treating the organic phase with saturated NaHCO_3_ solution five times, the effective transformation of the Cl^−^- to ${\text{HCO}}_{3}^{-}$-type quaternary ammonium salt can be realized, and the effective transformation of TOMAC from ${\text{HCO}}_{3}^{-}$ to ${\text{CO}}_{3}^{2-}$ type can be achieved by alkaline washing with 1.0 mol/l NaOH solution thrice. The extraction of thioarsenite by ${\text{CO}}_{3}^{2-}$-type TOMAC is conducted at an association molar ratio of 2:1, with the As^III^ saturated capacity in the loaded organic phase up to 0.21 mol/l.

The study investigated the influences of extraction of thioarsenious acid by ${\text{CO}}_{3}^{2-}$-type TOMAC, based on the volume fraction of ${\text{CO}}_{3}^{2-}$-type TOMAC, volume fraction of 2-octanol, temperature, time, and phase ratio. With the organic phase composition of “30% ${\text{CO}}_{3}^{2-}$-type TOMAC + 15% DL-2-Octanol + 55% sulfonated kerosene,” the single-stage As^III^ extraction rate reaches 85.2% with V_O_/V_A_ = 1/1 at room temperature for 5 min. After the four-stage countercurrent extraction, the concentration of As^III^ in the raffinate can be reduced to less than 1.33 × 10^−3^ mol/l, and the extraction rate of As^III^ can reach greater than 98.4%. Hence, the extraction of thioarsenite by ${\text{CO}}_{3}^{2-}$-type TOMAC can serve as an alternative for the removal of arsenic via alkaline leaching from high arsenic flue dust produced by heavy metal smelting.

## Data Availability Statement

The original contributions presented in the study are included in the article/supplementary materials, further inquiries can be directed to the corresponding author/s.

## Author Contributions

KY: conceptualization, methodology, validation, formal analysis, investigation, writing - original draft, data curation, resources, writing – review, and editing. LL: methodology, validation, formal analysis, investigation, writing - original draft, data curation, writing – review, and editing. HZ: formal analysis. LT: writing - original draft, data curation, writing – review, and editing. ZX: supervision, project administration, resources, funding acquisition, writing – review, and editing. RW: supervision, writing – review, and editing. All authors contributed to the article and approved the submitted version.

## Conflict of Interest

The authors declare that the research was conducted in the absence of any commercial or financial relationships that could be construed as a potential conflict of interest.
